# Reflection of therapy progress in virtual reality for individuals affected by obesity: a pilot study

**DOI:** 10.1007/s40519-025-01780-x

**Published:** 2025-09-17

**Authors:** Tatjana Anne Korbanka, Sandra Schild, Isabelle Mack, Katrin Elisabeth Giel, Simone Claire Behrens

**Affiliations:** 1 Medical Clinic, Psychosomatic Medicine and Psychotherapy, University Hospital Tübingen, Osianderstr. 5, 72076 Tübingen, Germany; 2 Centre of Excellence for Eating Disorders (KOMET), Tübingen, Germany; 3 German Center for Mental Health (DZPG), Tübingen, Germany; 4 Max Planck Institute for Intelligent Systems, Tübingen, Germany

**Keywords:** Obesity, Virtual reality, Psychotherapy research, Cognitive therapy

## Abstract

**Purpose:**

Obesity is a major health challenge, requiring the development of more effective interventions. Setting realistic goals and developing detailed plans that anticipate potential challenges can be helpful strategies for bridging the intention–behavior gap. Immersive technologies, such as virtual reality (VR), are considered to further support closing this gap by reducing the distance to one's future self.

**Methods:**

This study investigates a novel VR-supported reflection exercise as an additional treatment module within a conservative obesity treatment regime. It examines its feasibility, acceptance and short- and medium-term effects on participants' therapy motivation, body dissatisfaction, self-esteem, depressive symptoms, and eating behavior. 23 participants (BMI: *M* = 43.44 kg/m^2^, *SD* = 4.42) were presented with a real-time animated body avatar that had the average body shape for their individual height, initial weight, and realistic target weight (− 10%), and standard appearance matched in terms of hair and skin color. The avatar could be viewed from first-person and mirror perspectives. The exercise included reflective questions about their experience, well-being, daily life, and eating and movement behavior.

**Results:**

Participation and dropout rates of the VR-supported reflection exercise, user experience ratings, assessments on general discomfort and qualitative feedback demonstrated good feasibility and acceptance of the VR exercise. There were no measurable effects of a single session on clinical outcomes, including motivation to change, eating disorder psychopathology, self-esteem and depressive symptoms.

**Conclusions:**

The VR-based reflection exercise was feasible and well-accepted. The sample was highly burdened with multimorbidity, severe obesity (grade III), and psychological stress. A clinical trial with sufficient dosage would be required to infer about clinical effects.

**Level of evidence:**

Level 4.

**Supplementary Information:**

The online version contains supplementary material available at 10.1007/s40519-025-01780-x.

## Introduction

Combating obesity remains one of the greatest challenges of the twenty-first century. Over the past three decades, the global prevalence of obesity has more than doubled, with more than 1 billion people affected in 2022 [1]. Obesity is associated with a range of somatic and psychological comorbidities, is one of the strongest risk factors for health impairments and can contribute to reduced life expectancy [2].

Obesity is considered a multifactorial condition with biological, social, and psychological determinants [2]. Treatment of obesity aims to reduce weight comorbidities, through lifestyle interventions aiming at behavioral modification, which may be supplemented by bariatric surgery or medication. Despite these treatment options, rapid weight regain is often reported after successful initial weight loss, accompanied by low self-efficacy expectations of those affected. To support long-term weight maintenance, strategies focusing on behavior modification, particularly by enhancing motivation for change, are recommended. A systematic review highlights that key aspects of behavioral change, such as self-monitoring and self-efficacy, are predictive of both weight management and improvements in health-promoting behaviors [3].

The intention–behavior gap focuses on this behavioral level, and can contribute to explaining insufficient weight loss and frequent weight regain within a health psychology approach. It postulates that, although individuals have the intention to change their behavior, they can lack the volitional factors to effectively translate these intentions into behavior [4]. For individuals with obesity, this means that while they intend to lose weight, they frequently struggle to implement the respective actions consistently and sustainably in their daily lives. This often leads to weight regain. To reduce this gap, theoretical models of behavior modification emphasize the importance of setting realistic and specific goals and developing detailed plans that anticipate potential challenges throughout the entire behavior change process [5]. Thus, a guided reflection on specific goals and the development of detailed plans should help individuals to modify their behavior.

Virtual reality (VR) is a promising approach to bridge the intention–behavior gap by reducing the psychological distance between individual’s current and future selves [6]. The immersive nature of VR, combined with its ability to induce a sense of presence, interact naturally with the environment, and induce embodiment of virtual avatars, can generate intensive experiences. These experiences can closely resemble those in the real world [7]. Body avatars represent an emerging trend in VR research and enable the experience of realistic and specific goals such as successful weight loss. Still, their application to individuals with obesity remains underexplored [8]. Existing VR studies on obesity are reported to be well-received in terms of acceptability and feasibility, but no participation rates were reported [8]. These studies have largely focused on simulating naturalistic scenarios to practice desired behaviors, often as stand-alone interventions. For example, VR has been used to immerse individuals with obesity in potentially stressful situations, such as grocery shopping. By doing so, neither a specific psychotherapeutic approach was pursued, nor were these experiences integrated into the broader therapeutic concept [8]. VR interventions that were conducted as part of conservative weight-loss programs were mainly take-home or web-based, rather than psychotherapeutically guided in an individual setting. As a result, the potential of VR interventions to bridge the intention–behavior gap for individuals with obesity remains largely untapped.

The present study seeks to advance VR interventions for effective obesity treatment piloting a VR-supported adjunct treatment to a conservative weight-loss program. Within a VR-supported reflection exercise addressing current and realistic target weight, volitional factors to bridge the intention–behavior gap are addressed by prompting realistic and specific goals and reflecting health-promoting plans. The already piloted VR environment [9] features a real-time animated biometric avatar of participants’ initial weight and a realistic target weight (guideline-compliant weight loss of − 10%). The study investigated (1) feasibility and acceptance of the VR-based reflection exercise within participants of a conservative weight-loss program; and (2) short- and medium-term effects of the VR-based reflection exercise on motivation for change, eating disorder symptoms, self-esteem, and depressive symptoms in individuals affected by obesity.

## Materials and methods

The study was pre-registered (https://aspredicted.org/M4V_BC9) and approved by the Ethics Committee of the Medical Faculty of the University Hospital Tübingen (No. 388/2023BO2). Before participating in the study, all participants provided written informed consent.

### Sample

Participants were recruited via the conservative weight-loss program VIADUKT I^1^ at the University Hospital Tübingen, Germany. Conservative management of obesity involves non-surgical and non-medical treatments aimed at sustainable lifestyle modification. Conducted in a group setting, VIADUKT I combines nutritional counseling, exercise therapy and elements of behavioral therapy, including strategies for increasing motivation for change. It is specifically tailored for individuals with obesity grade III—those who are severely affected and seek support either because they wish to avoid bariatric surgery or because they have not yet fully exhausted conservative therapeutic options. It comprises ten group sessions over 6 months, occurring biweekly. Recruitment for the study took place during the fifth or tenth session of the VIADUKT I courses (interim evaluation on learning progress or final session of the program). This was done to ensure a certain homogeneity in terms of progress in the program and to recruit as many participants as possible.

Inclusion criteria were current or former participation in VIADUKT I, a minimum age of 18 years and sufficient knowledge of German. Exclusion criteria were diagnoses of an acute psychotic, bipolar or body dysmorphic disorder, alcohol or other substance dependence or suicidal tendencies. No reimbursement was provided.

### Reflection exercise and procedure

The VR setup used was previously tested in two pilot projects involving women with severe weight and figure concerns, as well as women with Anorexia nervosa, and was shown to be user-friendly [9]. The VR setup consists of a wired head-mounted display (HMD) (HTC VIVE Pro) connected to a gaming laptop, along with two Steam Valve controllers for hand tracking and four VIVE trackers to monitor the positions of the participants' upper arms and ankles. Two SteamVR base stations were positioned in front of the participant. For detailed information on the VR setup see Behrens et al. [9].

The VR environment shows a U-shaped cabin designed to simulate a changing room. The participant is represented by a full-body avatar positioned at the center of the cabin. It can be viewed in full-body size from head to toe in front of a frontal mirror, a mirror on the left side and from a first-person perspective. The avatar’s face is obscured by a 3D model of an HMD. The skin and hair color of the avatar are closely matched to the participant's actual skin and hair colors using textures from the BEDLAM project [10].

**Fig. 1:**
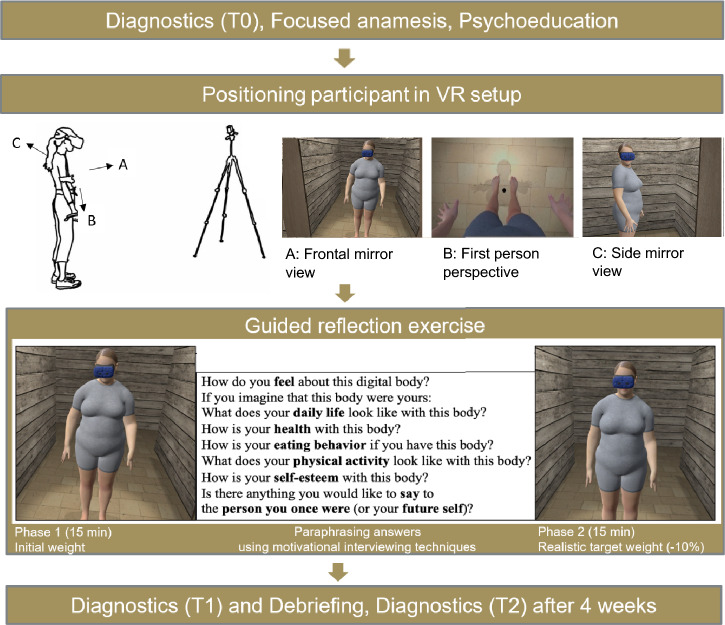
The 30-min reflection exercise led by the therapist was carried out in person in an individual setting. Participants revisited and reflected on key topics from VIADUKT I that they personally raised (e.g., the energy density of food). They engaged with this knowledge by reflecting on how they implement it with their initial weight (phase 1) or realistic target weight (phase 2). Using motivational interviewing techniques, the questions of the reflection exercise were adapted to the discussed topics in the previous anamnesis (T0). Questions in phase 2 were adapted to include narratives from phase 1. A study manual was used to ensure that the procedure was as standardized as possible (see supplements)

There were three measurement times (T0, T1, T2). T0 was conducted prior to the reflection exercise and involved diagnostic assessments, along with the collection of health-related information. This included information on physical illnesses, current or past mental disorders, ongoing psychotherapeutic treatment and medication intake, all of which were gathered through open-ended questions. T1 took place immediately after the reflection exercise and involved diagnostic assessments as well as open-ended questions regarding possible side effects due to the use of the VR setup, the likelihood of recommending the use of the HMD to others affected, and the impressions and experiences from participating in the study. T2 took place 4 weeks after the study appointment and involved diagnostic assessments. For detailed information on the study procedure and reflection exercise, see Fig. 1.

### Statistical analysis

Hypotheses were tested at a significance level of *α* = 0.05. Descriptive statistics were calculated, and multiple one-way ANOVA with repeated measures (T0, T1 and T2) were performed. In addition, free-response comments were summarized by the author.

## Results

The study sample consisted of *N* = 23 participants (*N* = 17 female, *N* = 6 male). At T0, one participant (4.35%) was classified as having grade I obesity, three (13.04%) had grade II obesity, and 19 (82.61%), were classified as having grade III obesity. Physical illnesses were reported by 15 participants (65.22%), with the most mentioned conditions being high blood pressure (*n* = 8) and hypothyroidism (*n* = 6). A total of 14 participants (60.87%) reported the regularly intake of medication. Mental disorders were reported by eight participants (34.78%), with the most common being major depression (*n* = 5) and panic disorder (*n* = 3). Three participants (13.04%) were undergoing psychotherapeutic treatment at the time of the study appointment.

The participation rate in the VR-supported reflection exercise was 40%. The study was presented in eleven VIADUKT I courses, each with four to six participants (total *N* = 55). A study appointment was arranged with 27 participants (49.09%). Ultimately, 23 participants (40%) completed the study, while five (18.52%) withdrew the participation due to illness or other personal reasons. In addition, one former VIADUKT I participant (N = 1) independently contacted the study management, expressed interest in participating, and was subsequently included in the study. All enrolled participants completed the study entirely.

Regarding feasibility, results from the VR questionnaires indicate an overall highly positive user experience with the highest mean score on item “uninteresting–interesting”. Participants reported a moderate feeling of presence on average. Side effects were minimal, with the item “shoulder tension” having the highest mean score; however, this effect was reported as negligible on average. Detailed information on the results of the VR questionnaires and the short- and medium-term changes in the target variables as well as further information on the sample characteristics can be found in Table 1.

**Table 1:** Sample characteristics and results of the pre-registered analyses at T0, T1 and T2

	Variables	N	Answer range	T0			T1			T2			ANOVA (T0 vs. T1 vs. T2)			Norm values*
				Mean	SD	Range	Mean	SD	Range	Mean	SD	Range	*F*	*p*	η^2^	
Sample characteristics	Age	23		44.22	12.62	21–62										
	BMI (kg/m^2^)	23		43.44	4.42	33.81–54.20										
	Weight (kg)	23		123.78	15.66	93.50–151.60										
	Weight change since start of VIADUKT I (kg)	23		0.65	5.74	− 21.50 to + 7.60										
VR questionnaires	User experience in VR (UEQ-S total score)	23	− 3 to 3				2.20	0.70	0.5–3.0							​​ < − 0.8 = negative − 0.8 to 0.8 = neutral > 0.8 = positive 1.5–2 = very good**
	Presence in VR (German IPQ total score)	23	0 to 6				3.09	1.05	1.21–4.79							see above
	General discomfort in VR use (SSQ total score)	23	1 to 4				1.31	0.28	1.0–1.86							
Target criteria	Motivation to change (URICA-S)	23	0 to 4	2.33	1.39	− 0.75 to 5.25	2.33	1.13	− 1.0 to 4.25	2.23	1.48	− 1.25 to 4	(2, 44) = 0.12	0.887	0.00	
	Eating disorder psychopathology (EDE-Q total score)	23	0 to 6	2.66	0.93	0.99–4.16				2.72	1.04	0.80–4.67	(1, 22) = 0.25	0.624	0.00	1.95–2.79***
	Self-esteem (RSES total score)	23	0 to 3	20.04	6.23	0–29	21.00	5.42	10–29	20.22	6.84	0–30	(2, 44) = 0.72	0.492	0.00	< 15 = low 16–19 = medium 20–30 = high
	Depressive symptoms (PHQ-8 total score)	23	0 to 3	7.74	4.81	1–22	7.17	3.59	1–13	7.83	4.88	1–23	(2, 44) = 0.86	0.430	0.00	5–9 = mild 10–14 = moderate 15–19 = moderately severe 20–24 = severe

T0: Prior to the reflection exercise. T1: Immediately following the reflection exercise. T2: Four weeks after the study appointment (T0 and T1). URICA-S, University of Rhode Island Change Assessment-Short, higher score indicates increased readiness and motivation to change behavior; EDE-Q, Eating Disorder Examination Questionnaire, higher values indicate higher eating disorder psychopathology; RSES, Rosenberg Self-Esteem Scale; PHQ-8, Patient Health Questionnaire-8; UEQ-S, User Experience Questionnaire-Short, higher values indicate a more positive user experience; German IPQ, German Igroup Presence Questionnaire, higher values indicate a greater sense of presence in VR; SSQ, Simulator Sickness Questionnaires, a modification of the questionnaire was used in which higher values indicate more pronounced side effects. Questionnaires were presented in German

*Respectively 90.-95. Percentile

**Adapted from Chen et al., 2020

***Given the high proportion of women and the average age of this sample, it is classified as part of the German norm sample of women aged ≤ 44 years

In free-response comments, *n* = 2 (8.7%) participants stated that they were affected by certain side effects, *n* = 21 (91.3%) denied this statement. Regarding participants’ impressions and experiences with the VR-based reflection exercise, the most frequently mentioned topics were motivation to lose weight (*n* = 6), visualization of the realistic target weight (*n* = 5) and positive experience (*n* = 4). In addition, *n* = 19 (82.61%) participants stated that they would recommend the use of VR to other affected people.

## Discussion

To our knowledge, this study is the first to use an animated biometric avatar to conduct a psychotherapeutic reflection exercise integrated into a conservative weight-loss program for patients with obesity. Acknowledging the innovative therapeutic concept and complex technical design, we focused on acceptance and feasibility of the intervention, aiming to inform a larger clinical study. The VR-supported exercise showed good acceptance and feasibility by those affected, as indicated by participation and dropout rates, user experience ratings, assessments on general discomfort and qualitative feedback. No short- or medium-term effects in clinical outcomes including motivation to change, eating disorder psychopathology, self-esteem and depressive symptoms could be found.

The VR-based reflection exercise was well-accepted, indicated by the participation rate of 40% of VIADUKT I participants that were invited to the study. A comparison with other VR studies on obesity is limited, because no participation rates were provided [8]. Nevertheless, alongside with the absence of dropouts, the high burden of the investigated sample and the lack of reimbursement for study participation, this participation rate can be interpreted as an indicator of high acceptance. This demonstrates that VR is suitable for harnessing the interest and willingness of patients with obesity to engage in psychotherapeutic interventions.

Overall, the exercise proved to be feasible for this highly burdened sample of obese participants. The subjective user experience of the VR environment can be assessed as very good, with a high level of interest reported by participants. The feeling of presence was assessed as neutral on average, which seems sufficient to conduct a VR-based reflection exercise of this kind. The general discomfort when using the HMD can be classified as low overall, with only occasional occurrence of side effects such as tension in the shoulders or back pain and the uncomfortable feeling of having to stand in the same place all the time. The latter could not be avoided due to the standing situation presented in VR. However, attaching foot trackers in future studies could allow participants to move more freely, thereby further increasing user comfort. Apart from this, no side effects were reported and most participants stated to fully recommend using the HMD, emphasizing the high experiential value and interesting nature of the study.

Free-response comments indicate that the VR-based reflection exercise can be beneficial for individuals with obesity. Some participants reported a perceived increase in motivation, with some indicating that the new body weight felt more tangible and that they would remember the associated feelings. In addition, a few participants mentioned that they could view a 10% weight loss as a potentially realistic goal. This suggests that VR-supported reflection may be a strong motivator to engage in lifestyle modification and, therefore, has great potential to support the therapeutic process.

Although our data collection was conducted in addition to ongoing treatment, there were no short- or medium-term changes in motivation to change, eating disorder symptoms, self-esteem or depressive symptoms, with effect sizes being minimal. This is an expectable finding given that our sample was, consistent with the literature [2], highly burdened: the studied sample showed pronounced mental strain and physical illnesses. Notably, the average BMI corresponded to obesity grade III, with most participants falling into this category. It is at hands that the one-time dosage of the 30-min VR-based reflection exercise in addition to ongoing treatment may have been insufficient for this burdened sample. This suggests a potential need for a higher dosage and further optimization of the procedure, with this study offering valuable insights for such improvements.

## Limits and strengths

An attempt was made to create the body avatar overall realistic and partly individualized by adjusting the skin and hair color of the body avatar to match participants' actual appearances. Still, it could not fully reflect their true looks, such as individualized body shapes or ages, potentially affecting the feeling of presence in the VR environment. Furthermore, while we asked about key VR aspects such as the feeling of presence, the study did not assess participants' experience of embodiment, limiting our ability to draw definitive conclusions about the extent of embodiment with the virtual body avatar. Despite our results suggesting that such a VR-supported reflection exercise can be a powerful motivator for lifestyle change in the therapy process, we were unable to clarify whether VR provided any added value over traditional imagination exercises. Determining this is crucial for assessing the cost-effectiveness of VR implementation and represents a key target for future studies. As identifying clinical effects was not the primary objective of this pilot study, the sample was underpowered to detect effects in this burdened group, making these results preliminary. Finally, the sample consisted of individuals who opted for a conservative treatment approach. Consequently, the acceptance of such VR interventions among those choosing bariatric surgery or medication remain to be determined.

Despite these limitations, the findings suggest that this innovative approach could serve as a feasible and acceptable tool to engage and motivate those affected by obesity for lifestyle modifications within a psychotherapeutic setting. Our VR implementation was both portable and quick to deploy, yet sufficient in creating a sense of presence and avatar identification. Further research is needed to optimize the technical aspects, dosage, therapeutic procedures, and to explore underlying mechanisms. Ensuring therapeutic involvement, ideally under close supervision and in consultation with the respective weight reduction program, and tailoring these interventions to personal needs through an individual setting seem to be crucial aspects. In addition, changes in self-efficacy of participants should be assessed, as these appear to be key variables for therapeutic success.

Clarifying the added value of VR over traditional imagination exercises, ideally with a powered sample, is an important next step.

## What is already known about this topic?

Sustainable weight loss remains a challenge in the treatment of obesity. There is consensus that patients need to make profound lifestyle modifications, but many have unrealistic weight goals and, therefore, struggle to implement effective measures to achieve their goals. Virtual embodiment of a healthy self has been proposed as a means of overcoming this distance and has already been piloted in patients with Anorexia nervosa and in single cases.

## What does this study add?

This study tests feasibility and acceptance of a manualized virtual reality supported add-on treatment module for patients with obesity that aims to support realistic weight goals and the implementation of behavior modifications. Data from 23 patients demonstrate that the use of virtual weight change through avatars in a guided reflection exercise for individuals with obesity is feasible, well-accepted and can be beneficial in terms of motivation and the acceptance of a realistic target weight.

## Supplementary Information

Below is the link to the electronic supplementary material.

Supplementary file 1.

## Data Availability

The data can be requested from the author via the following link: https://osf.io/jm9a4/?view_only=d559d22a3efd4dd5a8181c64af978797.

## Footnotes

1 https://www.medizin.uni-tuebingen.de/de/das-klinikum/einrichtungen/kliniken/medizinische-klinik/psychosomatik/ambulanz/viadukt-programme
