# Supplementary material for: Reflection of therapy progress in virtual reality for individuals affected by obesity: a pilot study
Source: Eat Weight Disord. 2025 Sep 17;30(1):74. doi: 10.1007/s40519-025-01780-x (PMC12443911; doi:10.1007/s40519-025-01780-x)
Supplement: Supplementary file 1 [file 40519_2025_1780_MOESM1_ESM.pdf]

Study Manual: Reflection of Therapy Progress in Virtual Reality for Individuals Affected by Obesity: A Pilot Study.

Tatjana Anne Korbanka[1,2,3], Sandra Schild[1,2], Isabelle Mack[1,2], Katrin Elisabeth Giel[1,2,3], Simone Claire Behrens[1,2,3,4]

1 University Hospital Tübingen, Medical Clinic, Psychosomatic Medicine and Psychotherapy, Tübingen, Germany

2 Centre of Excellence for Eating Disorders (KOMET), Tübingen, Germany

3 German Center for Mental Health (DZPG), Tübingen, Germany

4 Max Planck Institute for Intelligent Systems, Tübingen, Germany

Corresponding Author:

Tatjana Anne Korbanka

Psychosomatic Medicine and Psychotherapy, Medical University Hospital

Osianderstr. 5

72076 Tübingen, GERMANY

Tel: 0049 7071 2980166

E-mail: tatjana.korbanka@med.uni-tuebingen.de

Welcome & Introduction

- “Welcome to our study. I already told you a bit about the study in your VIADUKT appointment, but I would like to describe the exact procedure of the study to you here.”
- “The main part of today's appointment is that we want to use VR glasses to simulate two different weights, your starting weight before the VIADUKT program and your target weight. This will allow you to feel your way into the realistic target weight and compare it with your starting weight.”
- “About today's process: I will collect the signed declaration of participation in a moment. Then we will go through a checklist to see if you meet all the criteria for participating in the study. I will then take a brief medical history, during which I will ask questions and we will record a few body-related measurements. You will then answer a few questionnaires on your laptop, which will take about 15 minutes. Then the main part of the study begins. I will explain the background to the reflection exercise. During the reflection exercise, I will then ask you questions about your thoughts and experiences. We will then debrief the exercise. At the end, you will fill in a few more questionnaires.”
- “The appointment is now scheduled for a total of 90 minutes. You can always say if you need a short break, need to go to the toilet or want something to drink.”
- “Do you have any questions so far?”

VP information and data protection sheet

- 2 signatures: Consent to participate and consent to data processing
- Collect the signed document

Generate VP code

- Hand out document, have VP code generated, collect

Checklist for participation in the study

- Complete the sheet “Checklist for participation in the study Reflection of therapy progress in virtual reality in patients with obesity - a pilot study (VR-Reflect)”

Medical history form

- Complete the sheet “Medical history form and body measurements for the study Reflection of therapy progress in virtual reality for patients with obesity - a pilot study (VR-Reflect)”

Oral Anamnesis

- When did you start the VIADUKT program, and where do you currently stand?
- What were your reasons for starting the VIADUKT program?
- What have you achieved so far? What were your key insights? Has your weight changed?
- We often hear that overweight patients strongly define themselves by their weight, meaning their self-esteem rises and falls in relation to their weight. How is that for you?

Questionnaires

- Prepare a loaner laptop and open the questionnaire windows.
- Pre-questionnaires (T0)
- During this: Set weight, height, and appearance on the dummy. (Note: Adjust the appearance separately for women and men.)

Weighing & Measuring

- Record data on the anamnesis form.

Psychoeducation

"Now, I would like to tell you a bit about the background of the reflection exercise, our approach, and the psychological processes behind it."

"The typical weight trajectory for people with obesity looks like this (draw a curve with an upward trend). After weight-loss programs, it often decreases, but over time, it tends to rise again (illustrate this). Many people struggle to maintain their weight after successful weight loss and fall back into old habits. Have you experienced this as well?"

"It is challenging to implement many small lifestyle changes in everyday life. Additionally, progress can be slow and hard to notice."

"Our goal is to help you achieve a trajectory like this (draw a long-term weight reduction). That is why it is important to pause and reflect on the steps you have already taken in the VIADUKT program and those still ahead. That is our focus today. We will emphasize all the changes—both visible and invisible—that you have made. Sometimes, there is a contrast between external, visible changes and the internal transformations you feel within yourself. We will discuss that as well."

"Today, we will create a body dummy at your target weight to make your goals feel more realistic. This can be very helpful in shifting the way you think about yourself."

"We will start with your initial weight and then look at what you can achieve if you stay committed. We will use a 10% weight reduction as a reference because it is both realistic and medically significant."

"Do you have any questions about this, or is anything unclear?"

Reflection Exercise

"I would like to invite you to mentally revisit the beginning of the VIADUKT program. I am curious about your thoughts—who you were back then and how you have changed since. To help with this, I have brought these devices. They will allow us to simulate your body weight at the start of the VIADUKT program. It may not look exactly like you, but this method has proven effective in various contexts for helping participants reconnect with their past experiences."

"Do you have any questions before we begin?"

"To orient yourself: You can look straight ahead as usual, but you can also look down at yourself. On your left side, there is a mirror. You can move your arms, look down at your body, and rotate your torso. I will give you a minute to settle into this body. Imagine this is your body now."

<u>Guided Questions:</u>

- How do you feel in this digital body?
- If this were your body, what would your daily life be like?
- How is your health in this body?
- What is your eating behavior like in this body?
- What is your movement behavior like in this body?
- How is your self-esteem in this body?
- How would you handle situation XXX (based on key insights from earlier) in this body?
- Before we leave this scenario: Is there anything you would like to say to your past self?
- (In the second round): Is there anything you would like to say to your future self?

"Thank you. I would like to leave it at that and bring you back to the present."

Debriefing

- How was the exercise for you?
- What aspects became clearer through the reflection exercise? Were there any new insights?
- What do you take away from today's session?

Post-Questionnaires

Complete the post-questionnaires (T1)

Closing

"Very well, that concludes today's session."

"I will send you a link via email in 3–4 weeks with additional questionnaires that you will need to complete. Please fill them out by XX.XX.2023. All details will also be included in the email."

"Thank you very much for your participation! If you have any questions or concerns, feel free to contact me via email. You already have my contact details."
